# Low c-Met expression levels are prognostic for and predict the benefits of temozolomide chemotherapy in malignant gliomas

**DOI:** 10.1038/srep21141

**Published:** 2016-02-16

**Authors:** Ming-Yang Li, Pei Yang, Yan-Wei Liu, Chuan-Bao Zhang, Kuan-Yu Wang, Yin-Yan Wang, Kun Yao, Wei Zhang, Xiao-Guang Qiu, Wen-Bin Li, Xiao-Xia Peng, Yong-Zhi Wang, Tao Jiang

**Affiliations:** 1 Department of Neurosurgery, Beijing Tiantan Hospital, Capital Medical University, Beijing, 100050, China; 2 Beijing Neurosurgical Institute, Beijing, 100050, China; 3 Department of Neurosurgery, First Affiliated Hospital of Dalian Medical University, Dalian Medical University, Dalian, Liao Ning Province, 116000, China; 4 Department of Pathology, Beijing Sanbo Brain Hospital, Capital Medical University, Beijing, China; 5 Department of Radiation Therapy, Beijing Tiantan Hospital, Capital Medical University, Beijing, China; 6 Department of Oncology, Beijing Shijitan Hospital, Capital Medical University, Beijing, China; 7 Department of Epidemiology and Biostatistics, School of Public Health and Family Medicine, Capital Medical University

## Abstract

Aberrant c-Met has been implicated in the development of many cancers. The objective of this study was to identify an unfavorable prognostic marker that might guide decisions regarding clinical treatment strategies for high-grade gliomas. C-Met expression was measured using immunohistochemistry in 783 gliomas, and we further analyzed c-Met mRNA levels using the Agilent Whole Genome mRNA Microarray in 286 frozen samples. *In vitro*, we performed cell migration and invasion assays. Cell sensitivity to temozolomide (TMZ) chemotherapy was determined using MTT assays. Both mRNA and protein levels of c-Met were significantly associated with tumor grade progression and inversely correlated with overall and progression-free survival in high-grade gliomas (all *P* < 0.0001). These findings were nearly consistent at the mRNA level across 3 independent cohorts. Multivariable analysis indicated that c-Met was an independent prognostic marker after adjusting for age, preoperative Karnofsky Performance Status (KPS) score, the extent of resection, radiotherapy, TMZ chemotherapy, and O6-methylguanine-DNA methyltransferase (MGMT) promoter methylation status. Further analysis *in vitro* revealed that downregulating the expression of c-Met dramatically inhibited cell migration and invasion capacities, enhanced sensitivity to TMZ chemotherapy in H4 and U87 glioma cells. Our results suggest that c-Met may serve as a potential predictive maker for clinical decision making.

Gliomas are the most frequent primary tumors of the central nervous system in humans with glioblastoma (WHO grade IV), which is the most common and malignant histological tumor type. Due to the heterogeneity and instability of cancer, the 5-year survival of patients with glioblastoma is <3%^1^, despite the use of multimodal therapies involving surgery, radiotherapy and chemotherapy. To date, a biomarker panel with predictive power to distinguish between treatment-sensitive and treatment-refractory gliomas is not available. Therefore, it is imperative to identify important markers, and ultimately, to target the mechanisms of these molecules. This approach will likely have a substantial influence on future treatment strategies for highly invasive gliomas.

Currently, it is widely accepted that the most consistent abnormalities associated with well-known molecular aberrations of aggressive gliomas, such as chromosome 7 amplification and chromosome 10 deletion (which were the primary concerns of our present study), are present in 80% to 90% of cases^2^,^3^,^4^. Recent developments in cytogenetic and molecular biology have provided new approaches for analyzing prognoses. Tumor suppressor genes, proto-oncogenes and markers of metastatic propensity and proliferation are some of the different research tools that have been used. Among these tools, the most interesting tumor marker is Met (a hepatocyte growth factor receptor), which is a transmembrane receptor protein that has been implicated in the pathogenesis of glioblastomas (GBMs) through autocrine and/or paracrine mechanisms that potentially affect tumor cell growth, survival, invasion, migration, and angiogenesis^5^,^6^,^7^,^8^. Hepatocyte growth factor (HGF) binding activates the tyrosine kinase of the Met receptor, which results in the auto-phosphorylation of several tyrosine residues in its cytoplasmic domain. The phosphorylation of each tyrosine residue initiates a distinct signal transduction cascade, and these cascades involve Erk, Akt, and Stat3^9^,^10^.

In humans, the c-Met gene is located on chromosome 7 at band 7q21–q31 and can be activated not only by binding HGF, its ligand, but also by the overexpression or amplification of factors that have been reported to occur in a wide variety of cancer types, including lung, stomach, liver, prostate, ovarian, colon, and pancreatic cancers^11^,^12^,^13^,^14^,^15^. In lung adenocarcinoma, immunohistochemical assessments of Met protein expression have revealed that Met is an independent predictor of a poor prognosis^16^, but its expression has not been revealed to be a prognostic and/or predictive marker of the effects of anti-MET/HGF therapeutics in a large high-grade glioma population.

Based on this background information, the objective of this study was to identify a molecular marker that can predict the effect of treatment and may be used as a prognostic marker for unfavorable outcomes in patients with highly diffuse astrocytomas. Therefore, we selected c-Met as the molecular target and evaluated the significance of the correlations of this marker with overall survival, progression-free survival and therapy outcomes in a large-scale study of high-grade tumor specimens.

## Materials and Methods

### Patients and Tumor Samples

A total of 885 patients with 486 grade II, 202 grade III, and 197 grade IV primary gliomas were included in the immunohistochemistry study. All of these patients were managed with surgical resection and subsequent chemoradiotherapy and were independently re-evaluated by 2 experienced neuropathologists who were blinded to the clinical outcomes of the patients, according to the World Health Organization 2007 criteria^17^. This study was approved by the Ethics Committee of Beijing Tiantan Hospital, the methods were carried out in accordance with the approved guidelines, and written informed consent was obtained from all patients included in this study.

### Treatment

Maximal tumor resection while preserving the key eloquent cortex was the principle goal during surgery. The extent of resection was assessed on the postoperative enhanced MRI within 72 h and graded as gross total or subtotal resection. Patients subsequently underwent concomitant TMZ and RT or radiotherapy in addition to concomitant and adjuvant TMZ chemotherapy (RT + TMZ) or postoperative radiotherapy only (RT only). Postoperative adjuvant radiotherapy was routinely delivered to the patient within four weeks after surgery. The total dose was 54–60 Gy, which was divided into 30 daily fractions of 1.8–2 Gy each, and five fractions were administered per week. For the patients who received adjuvant chemotherapy, the treatment was administered four weeks after radiation, and at least two cycles of chemotherapy were administered. Concomitant chemotherapy consisted of oral TMZ at a daily dose of 75 mg/m^18^ that was given seven days per week from the first to the last day of radiotherapy for at most 49 days. After a four-week break, the patients received adjuvant oral TMZ (150–200 mg/m2) for five days every 28 days. A total of six cycles of TMZ chemotherapy were administered if no disease progression or irreversible hematological toxic effects were observed.

### Surveillance and follow-up

Survival data were collected in the clinics during the patient visits. The patients who underwent only tumor biopsy were not followed up at our center and were therefore excluded from the survival analysis. The baseline examinations included CT and magnetic resonance imaging (MRI), full blood counts and blood chemistry tests and physical examinations. During radiotherapy (with or without TMZ), the patients were seen every week. Twenty-one to 28 days after the completion of radiotherapy and every three months thereafter, the patients underwent comprehensive evaluations that included physical examinations and radiologic assessments of the tumors. During the adjuvant TMZ therapy, the patients underwent monthly clinical evaluations and comprehensive evaluations at the end of cycles 3 and 6. Overall survival (OS) was defined as the time between surgery and death. Progression-free survival (PFS) was defined based on the RANO criteria^19^.

### Microarray Datasets

We used the whole genome mRNA expression microarray data for all tumor grades from Chinese Glioma Genome Atlas (CGGA) database^20^ (http://www.cgga.org.cn) as a validation platform. The US National Cancer Institute Repository for Molecular Brain Neoplasia Data (REMBRANDT, http://caintegrator-info.nci.nih.gov/rembrandt), GSE16011 Data^21^ (http://www.ncbi.nlm.nih.gov/geo/query/acc.cgi?acc=GSE16011) and the Cancer Genome Atlas (TCGA) Database^22^ (http://cancergenome.nih.gov) were used to validate the mRNA and protein sets.

### DNA extraction and DNA pyrosequencing for the IDH1 mutation and MGMT promoter methylation

All of the materials were selected for DNA extraction via an elaborate examination of corresponding hematoxylin and eosin-stained sections. Every selected sample contained at least 80% of the vital tumor and was quick-frozen in liquid nitrogen and maintained at −80 °C. Genomic DNA was extracted from the frozen tumor tissues using a QIAamp DNA Mini Kit (Qiagen) according to the manufacturer’s protocol. The genomic region spanning wild-type R132 of IDH1 was analyzed with pyrophosphate sequencing using the following primers: (forward) 5′-GCTTGTGAGTGGATGGGTAAAAC-3′ and (reverse) 5′-biotin-TTGCCAACATGACTTACTTGATC-3′. For O-6-methylguanine-DNA methyltransferase (MGMT) promoter methylation, bisulfite modification of the DNA was performed using an EpiTect Kit. The following primers were used to amplify the MGMT promoter region: (forward) 5′-GTTTYGGATATGTTGGGATA-3′ and (reverse) 5′-biotin-ACCCAAACACTCACCAAATC-3′.

### Cell lines and Cell Culture

*The human glioma cell lines H4 and U87 were purchased from the Institute of Biochemistry and Cell Biology, Chinese* Academy of Science. The cells were cultured in DMEM containing 10% FBS, 50 U/ml penicillin G and 250 μg/ml streptomycin in a humidified atmosphere containing 5% CO_2_ at 37 °C. H4 and U87 cells were transfected with c-Met shRNA (3 target-specific plasmids, GeneChem, China, MET-RNA1 (15908-1), MET-RNA2 (16973-1), and MET-RNA3 (16985-1) and a control vector) using Lipofectamine 2000 (Invitrogen) according to the manufacturer’s instructions. The MET-RNA1 target sequence was 5′-CTTATATGAAGTAATGCTA-3′, the MET-RNA2 target sequence was 5′-ACGATGAATACATTGAAAT-3′, and the MET-RNA3 target sequence was 5′-AACTTCTTTGTAGGCAATA-3′.

### Western blot

Whole cells were washed with cold PBS and lysed in RIPA buffer. Equal amounts of total protein (30 μg) from cell lysates were loaded onto 10% SDS/PAGE gels, transferred to PVDF membranes (Millipore), and detected using an ECL Western Blotting Detection System (Bio-Rad). The antibodies used were c-Met (1:2000 dilution, EP1454Y, Abcam) and GAPDH (1:5000 dilution, Proteintech).

### Cell Proliferation

For the MTT assays, the cells, including all transfectants, were grown to the exponential phase and detached by trypsin treatment. A total of 2000 cells/well were plated onto 96-well tissue culture plates (100 μl complete medium/well) and cultured at 37 °C in 5% CO_2_. Different TMZ concentration gradients were performed on H4 and U87 cells, and after 3 days, 20 μL per well of MTT reagent was added and incubated at 37 °C for 1 hour. Subsequently, the absorbance values of each well were measured with a microplate spectrophotometer at 490 nm. Results were plotted as means ± SD of three independent experiments.

### Cell migration and invasion assays

24-well chambers with 3.0 μm pore size (Corning) were used in cell migration and invasion assays. Cells were resuspended in 500 μL serum-free medium and 100 μL (about 100,000 cells) were seeded into the upper chamber (without or pre-coated with 200 ng/ml Matrigel solution (BD Biosciences, Bedford MA 01730, USA) in migration or invasion assay separately), 600 μL of DMEM containing 10% fetal bovine serum was placed in the lower chamber. After 24 h of incubation, the upper chambers were removed from the plates and cells on the top side of the chamber were wiped with a cotton swab. Migrating or invading cells were fixed and then stained. Four randomly fields were counted under a microscope and photos were taken.

### Immunohistochemistry (IHC)

Tumor samples collected during surgery or biopsy were embedded in paraffin and evaluated by experienced neuropathologists. One appropriate block from each specimen was selected, and serial 4 μm-thick sections of tissue were used for the immunohistochemical analyses of c-Met expression. In brief, immunoperoxidase staining for c-Met (Santa Cruz Biotechnology, sc-161) was performed following the standard protocol recommended by the manufacturer to show membrane and cytoplasmic staining. Each slide that was stained for c-Met was individually reviewed at 200x magnification and scored by two independent observers. Discrepancies in scoring between the two observers were resolved by an additional review of the specimens and a discussion was held between the reviewers until a consensus was achieved. The proportions of positively stained tumor cells were graded as follows: 0, no positive tumor cells; 1, <10% positive tumor cells; 2, 10–30% positive tumor cells; and 3, >30% positive tumor cells. The intensity of staining was recorded on the following scale: 0 (no staining), 1 (weak staining, light yellow), 2 (moderate staining, yellowish brown), and 3 (strong staining, brown). The staining index was calculated as follows: staining index=staining intensity×tumor cell staining grade. High c-Met expression was defined as a staining index score ≥4, and low expression was defined as a staining index <4.

### Statistical Analysis

Descriptive statistics, included counts, mean values, and standard errors, were calculated, as appropriate. The differences in c-Met expression were examined with ANOVA. The OS was calculated from the date of surgery to the date of the last follow-up or the date of death. The PFS was calculated from the date of the first surgery until the recurrence or the last follow-up. Survival curves were drawn using the Kaplan–Meier method, and the differences between the groups were compared with log-rank tests. P-values < 0.05 were considered statistically significant. Univariate analyses and multivariate analysis were performed with the Cox regression model. The factors that exhibited no significant association were eliminated (P > 0.05), and the remaining factors (P < 0.05) that were entered into the multivariate analysis were assumed to be independent predictors of survival. All data analyses were performed with GraphPad Prism, R and SPSS (Statistical Package for the Social Sciences) software.

## Results

### Patient Demographic Characteristics

For immunohistochemistry, we selected a patient population with available concrete clinical information that included 429 grade II, 179 grade III, and 175 pGBMs for our training set. The characteristics of patients with grade III and primary GBM are given in Table 1. Regarding the mRNA platform, among the data from the 286 glioma patients whose medical records were obtained from the CGGA database, there were 126 grade II gliomas, 51 grade III gliomas and 109 pGBMs. The other 264 and 317 samples from the GSE16011 and Rembrandt databases, respectively, were used as validation sets.

### MET was overexpressed in high-grade gliomas

**Figure 1:**
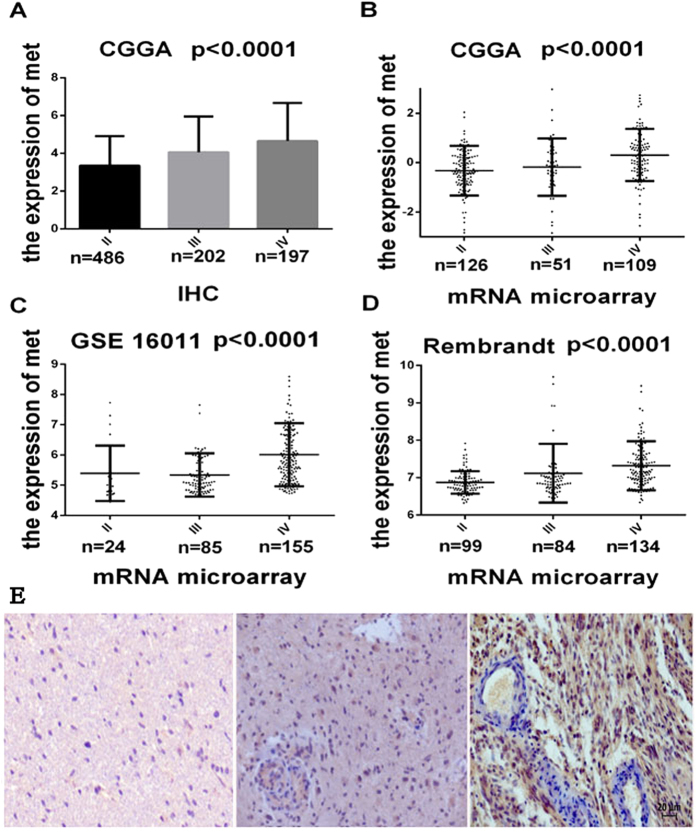
c-MET differences in all grades and all three databases. (**A**) The protein expression patterns of c-Met in the CGGA IHC validation cohort, which contained 887 glioma tissues. (**B**, **C**, **D**) c-Met expression patterns in the microarray validation cohorts, which contained 286 (CGGA), 317 (REMBRANDT) and 264 (GSE16011) glioma tissues. Single spots indicate the gene expression values of individual patients. The lines in the middle indicate the mean expression values. (**E**) Representative antibody staining for c-Met (Grade II-left, Grade III-middle, and pGBM-right) showing that c-Met expression increased gradually with increasing tumor grade. All slides stained for c-Met were individually reviewed at 200x magnification. Scalebar 20 μm. ANOVA was to examine the overall P values.

The expression of c-Met was measured in a series of 885 glioma samples by IHC (Fig. 1E). As shown in Fig. 1A, the pGBMs and anaplastic gliomas exhibited significantly higher levels of c-Met expression than the low-grade gliomas (P < 0.0001). To further confirm this result, the other 3 validation data sets were examined, and the microarray results revealed that the pGBMs exhibited a significant increase in c-Met transcript level compared with the mean expression levels observed in the low-grade gliomas and anaplastic gliomas (P < 0.0001; Fig. 1B–D).

### MET was a strong prognostic marker in the high-grade gliomas

**Figure 2:**
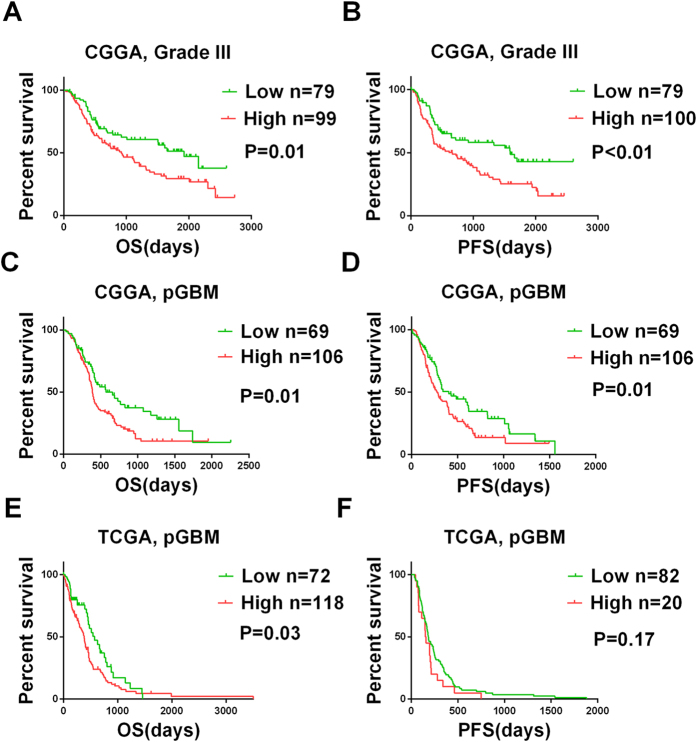
The prognostic value of the c-Met protein in the CGGA and validation datasets. Kaplan-Meier curves for the two high-grade glioma databases that were constructed according to the protein expression of c-Met. In the CGGA datasets, overall survival (**A**) and progression-free survival (**B**) show that grade III patients carrying negative c-Met protein expression survived significantly longer. Overall survival (**C**) and progression-free survival (**D**) show that pGBM patients carrying negative c-Met protein expression survived significantly longer. In the TCGA datasets, overall survival (**E**) and progression-free survival (**F**) show that pGBM patients carrying negative c-Met protein expression survived longer. The CGGA values were acquired via IHC, and the TCGA values were downloaded from the TCGA web site. The log-rank test was used to calculate the P values.

To achieve stratification based on c-Met protein expression, the patients were divided into high c-Met expression and low c-Met expression groups based on the staining indices. The mean overall survival times of the pGBM and grade III patients with high c-Met expression were obviously shorter than those of the corresponding patients with low c-Met expression (P = 0.01 and P = 0.01, respectively, by log-rank test; Fig. 2). The mean progression-free survival time of pGBM and grade III patients with low c-Met was significantly longer than that of patients with high c-Met expression (P = 0.01 and P < 0.01, respectively, by log-rank test; Fig. 2). Subsequently, these results were confirmed based on the mRNA levels in the three validation sets (Figure S1). To determine the prognostic value of c-Met in high-grade gliomas, we conducted univariate Cox regression analyses using the clinical and genetic variables of the high-grade patients. We found that age, preoperative KPS score, extent of resection, radiotherapy, chemotherapy, MGMT promoter methylation, and c-Met expression were statistically associated with the OS of the pGBM patients. Next, multivariable regression analysis was applied to evaluate the independent prognostic values of these clinicopathologic factors for patient survival. Ultimately, all of the data revealed that c-Met expression was an independent prognostic factor when age, preoperative KPS score, extent of resection, radiotherapy, chemotherapy, and MGMT promoter methylation were considered (Table 2). Unexpectedly, the same result was not observed in the grade III glioma patients (data not shown).

### Association of c-Met expression with the efficacy of currently available therapies

**Figure 3:**
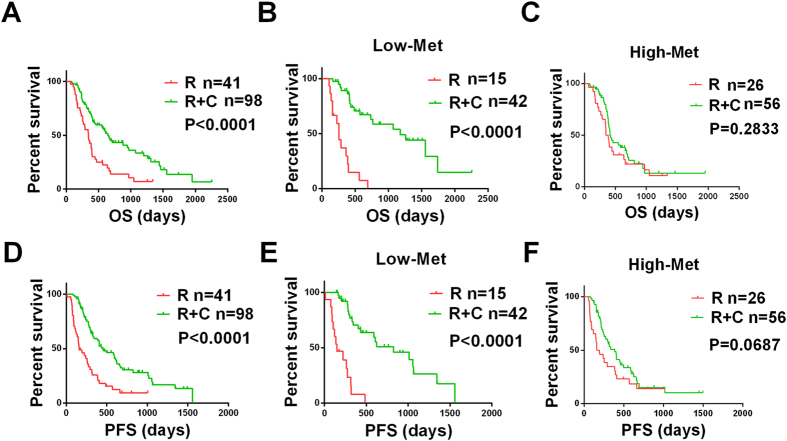
c-Met expression was correlated with treatment according to protein expression. Overall survival (**A**) and progression-free survival (**D**) of all of the pGBM patients who received combined chemotherapy and radiotherapy were longer than those who received only radiotherapy. The overall survival (**B**) and progression-free survival (**E**) of participants who received combined chemotherapy and radiotherapy and who displayed negative c-Met protein expression were longer. Overall Survival (**C**) and progression-free survival (**F**) of participants who received combined chemotherapy and radiotherapy and displayed positive c-Met protein expression were not significant longer than participants who received only radiotherapy. All values were acquired by IHC. R, radiotherapy; C, chemotherapy.

In our research, the patients who received aggressive therapy achieved the best OS and PFS, and the patients with pGBMs who received only radiotherapy exhibited the worst survival (Fig. 3A, D; All P < 0.0001). To further explain this phenomenon, we postulated an association between c-Met expression and therapeutic outcome and divided the patients into high-expression and low-expression groups and then further subdivided these groups into alkylating agent-treated and non-alkylating agent-treated subgroups. We found that the patients in the low-expression group were perfectly separated, which indicates that the patients who accepted aggressive therapy experienced longer OS (Fig. 3B, P < 0.0001) and PFS (Fig. 3E, P < 0.0001) than the patients who received only radiotherapy but that the same phenomenon was not observed in the high-expression group (Fig. 3C, P = 0.2833; Fig. 3F, P = 0.0687). The same method was also applied to the whole-genome mRNA expression microarray data and the same phenomenon was observed (Figure S2).

### c-Met sensitizes temozolomide (TMZ) therapy *in vitro*

**Figure 4:**
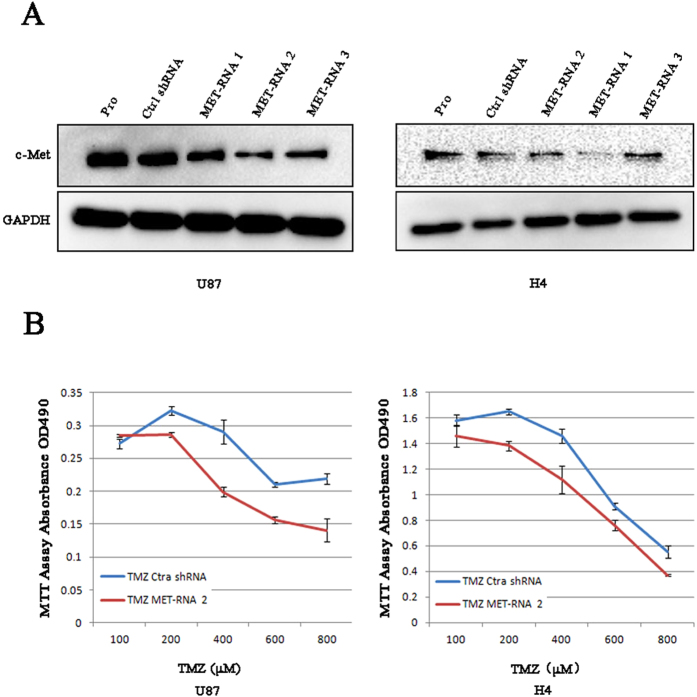
c-Met knockdown inhibits glioma cell proliferation *in vitro*. Western blot analysis showed the biological effects of 3 c-Met shRNAis. (**A**) The c-Met protein levels of U87 and H4 cells transfected with shRNA 1, one of 3 c-Met shRNAis, were nearly knocked down compared to control shRNA (Ctrl shRNA). *In vitro* growth of U87/Ctrl shRNA, U87/Met shRNA2 cells and H4/Ctrl shRNA, H4/Met shRNA2 cells were measured using MTT assays. (**B**) The proliferation of cells with low c-Met expression who received TMZ-treatment was clearly inhibited. The results represent at least 3 separate experiments. Bars, SD.

To underscore the potential for a medication-guiding role for c-Met, we first downregulated the expression of c-Met in two glioma cell lines that originally expressed c-Met at high levels. We followed this with a functional assay. Compared with control shRNA, MET-RNA2 clearly decreased the protein expression of c-Met (Fig. 4A), and H4 and U87 cells presented inhibited proliferation with TMZ-treatment in the absence of c-Met (Fig. 4B) according to an MTT assay, whereas application of c-Met with control shRNA had a reduced effect. The same result was also verified by analysis of mRNA and protein levels (Figure S3). Consequently, inhibiting MET enhanced the effect of TMZ sensitivity in both cell lines.

### Cell migration and invasion were decreased by c-Met-RNA2

**Figure 5:**
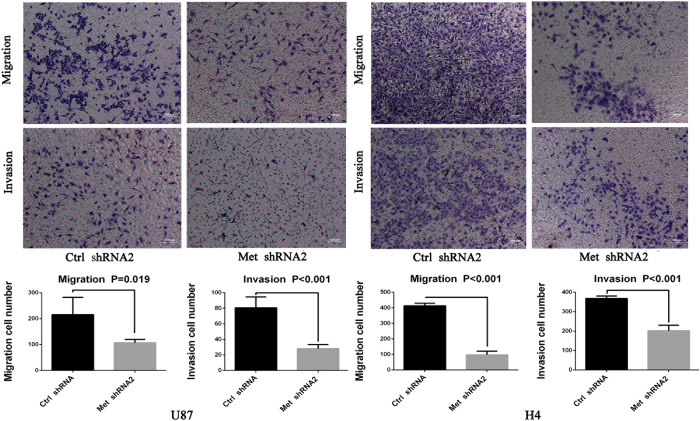
c-Met knockdown inhibits glioma cell migration and invasion. Quantification of migration and invasion cells showed that the cell migration and invasion capacities in c-Met-shRNA group were remarkably inhibited (P ≤ 0.01). Representative images and accompanying statistical plots were presented. Data are presented as the mean ± SD (n = 4, each group).

The migration assay and Matrigel invasion assay were performed using U87 and H4 cells. The migration assay showed that remarkable cell migration diminution in c-Met-shRNA group compared with in the control shRNA group (Fig. 5). The Matrigel invasion assay also showed numbers of invading cells in the control shRNA group were significantly increased compared with the c-Met-shRNA group (Fig. 5).

## Discussion

C-Met has been demonstrated to be expressed by various cell types, including epithelial cells, vascular endothelial cells, lymphatic endothelial cells, neural cells and hepatocytes^23^,^24^,^25^. Furthermore, c-Met is also overexpressed in multiple malignancies and is associated with aggressive disease^26^. Based on immunohistochemical analysis, Nabeshima *et al.* concluded that the expression of c-Met is correlated with the grade of malignancy in human astrocytic tumors^27^, Lee *et al.* found that the overexpression of c-Met is associated with the survival of patients with colorectal cancer^28^, and Kong *et al.* and Petterson *et al.* came to similar conclusions for patients who have GBMs^29^,^30^. Our findings that c-Met expression in the brain is associated with glioma progression and that among the patients with high-grade gliomas, those with high levels of c-Met expression exhibited shorter OS and PFS compared with the patients with low levels of expression strongly corroborate these previous results. Accordingly, it is also notable that our results expand on the previous results which included a larger and more ethnically diverse clinical population and are thus more persuasive. Further analyses indicated that the patients with low c-Met expression levels gained greater survival benefits from alkylating radiochemotherapy (P < 0.001 for OS and PFS at both the protein and mRNA levels) than did those with high c-Met expression levels (all P > 0.1). *In vitro* analysis revealed that the downregulation of the expression of c-Met dramatically enhanced sensitivity to TMZ chemotherapy in H4 and U87 glioma cells.

MGMT gene promoter methylation is well known to be a prognostic marker of favorable outcomes and is predictive of the benefits attained by alkylating agent chemotherapy in patients with GBM^31^. Accumulating experimental evidence indicates that constitutive activation of the P13K/AKT signaling pathway is associated with tumor cell resistance to conventional chemotherapy^32^,^33^, and Wang *et al.* noted that the effectiveness of TMZ is dramatically enhanced when STAT3 is inhibited^34^. However, no stronger molecular characterization of this malignancy had been identified in pGBM, so we identified c-Met as a robust marker with independent prognostic value for pGBM when considering much more important clinical information.

Highly diffuse glioma is a morphologically heterogeneous type of tumor for which the median survival has been reported to be only 15 months in clinical trial populations. Therefore, despite many advances in the treatment of these tumors, malignant gliomas remain the leading global cause of cancer-related deaths. Therefore, further research is needed to identify novel therapeutic targets for use in individualized treatment. In our samples, we focused on alkylating agent treatments and observed that the patients who received combined chemotherapy and radiotherapy experienced had longer survival than the patients who had only received radiotherapy. However, survival varied widely across patients. Therefore, we addressed the question of whether the patients with pGBMs displayed distinct biological features that responded differentially to combined chemo-radiotherapy and, if so, which biological mechanisms or genes were responsible for this phenomenon. Recently, Ozasa reported that c-MET overexpression might play an important role in acquired resistance to cytotoxic anticancer drugs^35^. According to this report, a potential therapeutic strategy aimed at inhibiting aberrant Met signaling is a promising therapeutic tactic for glioblastomas. In this study, we uncovered, for the first time in such a large-scale population, a previously unknown role for c-Met in gliomas. Our study revealed that patients with low levels of c-Met expression who received alkylating treatments had longer survival times and PFS than those who received non-alkylating treatment, but there was no significant difference in the high-expression c-Met group. To confirm these findings *in vitro*, we used c-Met knockdown via shRNA with TMZ treatments to inhibit glioma cell proliferation and compared results with Met-positive glioma cells, further supporting a role for downregulating expression of c-Met in temozolomide sensitivity. In order to further explore the crucial role of c-Met in the biological processes of tumor cell. We performed Cell migration and invasion assays which significantly revealed that c-Met-shRNA cells were obviously inhibited migration and invasion abilities of the glioma cells. Taken together, this study provided evidences that the decrease of c-Met expression was associated with prognosis and the biological functions of gliomas. Moreover, we found that high expression of c-Met might predict TMZ chemotherapy resistance both for patients and *in vitro*. Based on these results, c-Met may be a useful biomarker for identifying patients who should be candidates for more aggressive therapies such as c-Met targeted medicine.

## Additional Information

**How to cite this article**: Li, M.-Y. *et al.* Low c-Met expression levels are prognostic for and predict the benefits of temozolomide chemotherapy in malignant gliomas. *Sci. Rep.* **6**, 21141; doi: 10.1038/srep21141 (2016).

## Supplementary Material

Supplementary Information

Supplementary dataset 1

## Figures and Tables

**Table 1:** Clinical characteristics of 354 high-grade gliomas by IHC.

Characteristics	Grade III N = 179	Grade IV N = 175
Gender		
Female/male	79/99 (44%)	67/108 (38%)
Age		
Median (range)	42 (14–74)	51 (9–81)
KPS score		
Median (range)	80 (50–98)	80 (50–70)
Median OS		
(days, 95% Cl)	704 (1.4–3.2)	382 (1.0–2.2)
Median PFS		
(days, 95% Cl)	510 (1.6–3.6)	255 (1.0–2.1)
Extent of resection		
GTR (%)	43%	48%

Abbreviations: IHC, mmunohistochemistry; KPS, Karnofsky Performance Status; OS, overall survival; PFS, progression-free survival; GTR, gross total resection.

**Table 2:** Cox Hazard Regression Analysis of Clinicopathologic Factors and the MET for Overall Survival in pGBM (n = 175).

Variable	Univariate analysis			Multivariate analysis		
	HR	95% CI	*P*	HR	95% CI	*P*
Sex	0.811	0.554–1.188	0.282			
Age	1.020	1.005–1.034	** < 0.010**	0.975	0.946–1.005	0.103
IDH1 mutation	0.580	0.316–1.067	0.08			
MGMT promoter methylation	0.552	0.346–0.881	**0.013**	0.758	0.331–1.738	0.513
c-Met	1.640	1.115–2.412	**0.01**	2.389	1.126–5.072	**0.023**
KPS	0.973	0.955–0.991	** < 0.01**	0.962	0.938–0.988	** < 0.01**
Extent of resection	1.579	1.086–2.297	**0.017**	0.826	0.397–1.720	0.610
Radiotherapy	0.490	0.294–0.818	** < 0.01**	0.478	0.199–1.150	0.099
Chemotherapy	0.387	0.262–0.569	** < 0.01**	0.237	0.113–0.499	** < 0.01**

Abbreviations: pGBM, primary glioblastomas multiforme; IDH1, isocitrate dehydrogenase 1; MGMT, O6-methylguanine–DNA methyltransferase; KPS, Karnofsky Performance Status.
