# Supplementary material for: Low c-Met expression levels are prognostic for and predict the benefits of temozolomide chemotherapy in malignant gliomas
Source: Sci Rep. 2016 Feb 16;6:21141. doi: 10.1038/srep21141 (PMC4754763; doi:10.1038/srep21141)
Supplement: Supplementary Information [file srep21141-s1.pdf]

Supplementary Information

Low c-Met expression levels are prognostic for and predict the benefits of temozolomide chemotherapy in malignant gliomas

Ming-Yang Li, Pei Yang, Yan-Wei Liu, Chuan-Bao Zhang, Kuan-Yu Wang, Yinyan Wang, Kun Yao, Wei Zhang, Xiao-Guang Qiu, Wen-Bin Li, Xiao-Xia Peng, Yong-Zhi Wang and Tao Jiang

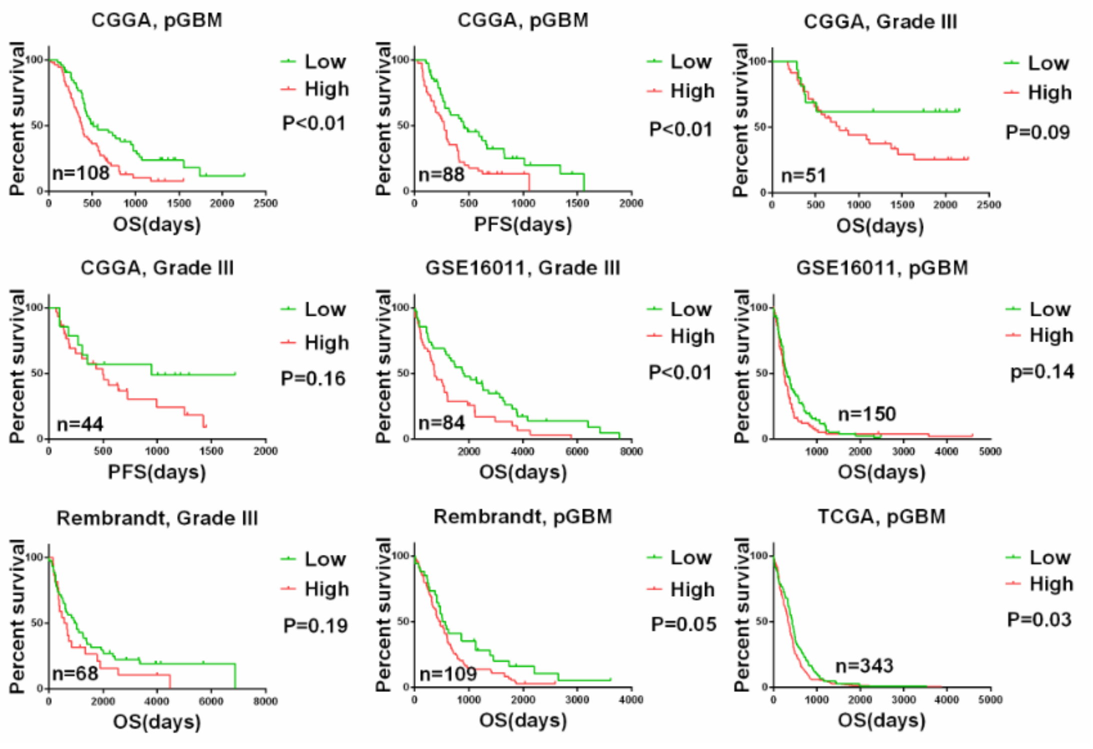

Figure S1. The prognostic value of c-Met in the CGGA and validation mRNA datasets. Kaplan-Meier curves showing the overall survival and progression-free survival of the different databases of high-grade gliomas that were constructed according to c-Met mRNA expression. Overall Survival and progression-free survival showed that high-grade gliomas carrying low c-Met mRNA expression were longer. The log-rank test was used to calculate the P values.

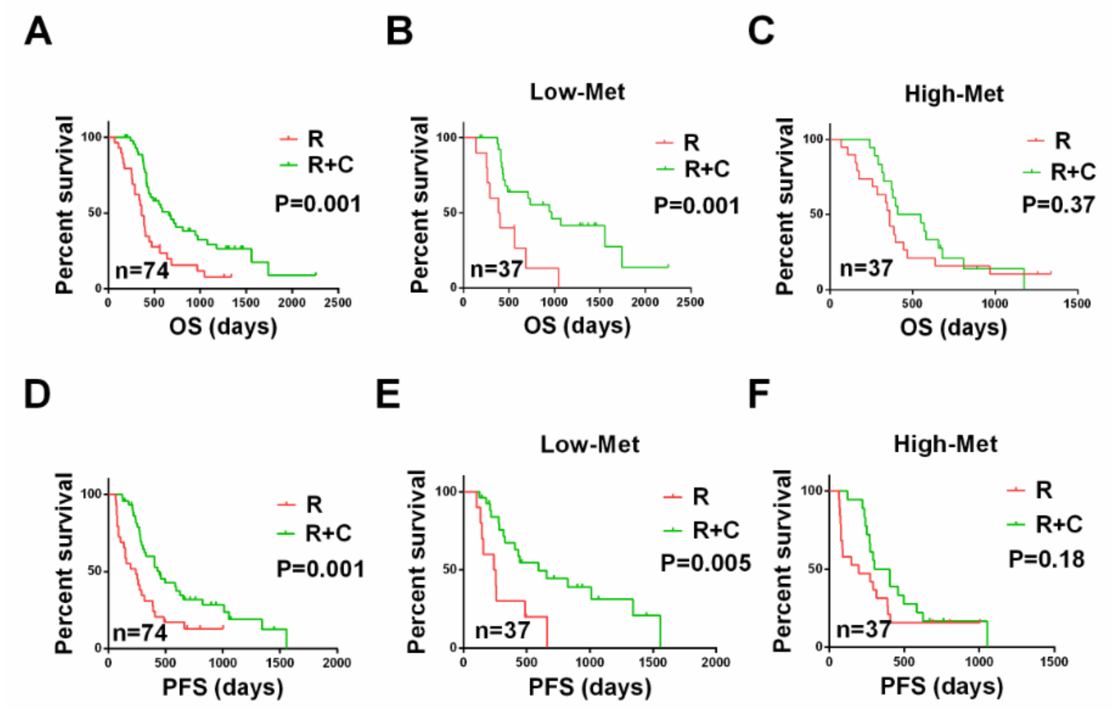

Figure S2. c-Met mRNA expression was correlated with treatment.

Overall survival (A) and progression-free survival (D) of all of the pGBM patients who received combined chemotherapy and radiotherapy had longer survival times than patients who received only radiotherapy. The overall survival (B) and progression-free survival (E) of participants who received combined chemotherapy and radiotherapy and displayed negative c-Met expression were longer. Overall survival (C) and progression-free survival (F) of participants who received combined chemotherapy and radiotherapy and displayed positive c-Met expression were not different than those of participants treated with only radiotherapy.

R, radiotherapy; C, chemotherapy.

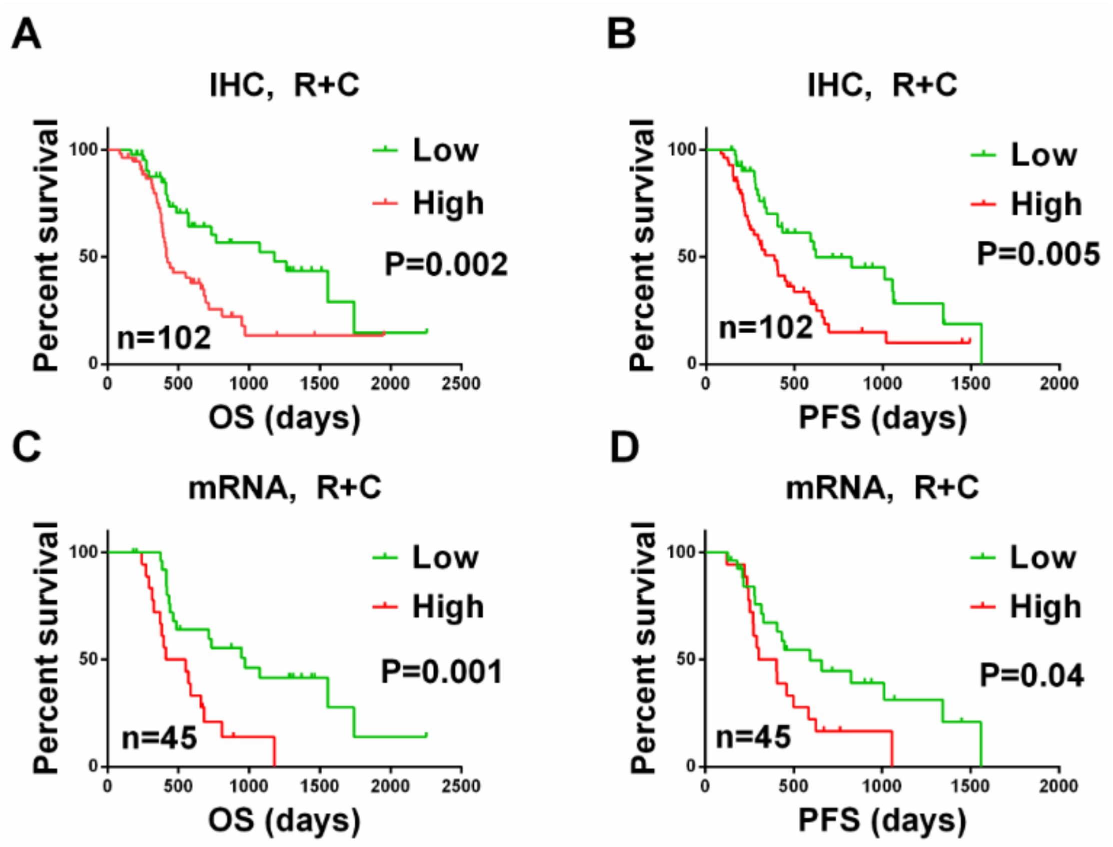

Figure S3. c-Met (at the mRNA and protein level) sensitizes temozolomide (TMZ) therapy

All participants received TMZ treatment. IHC results showing c-Met protein expression levels were analyzed by Kaplan-Meier analysis in glioma participants. The overall survival (A) and progression-free survival (B) of participants displaying negative c-Met expression were significantly longer. Kaplan-Meier analyses were performed to analyze differences in c-Met mRNA expression levels in glioma participants. The overall survival (C) and progression-free survival (D) of participants displaying negative c-Met expression were found to be longer.
